# A Novel Positron Emission Tomography (PET) Approach to Monitor Cardiac Metabolic Pathway Remodeling in Response to Sunitinib Malate

**DOI:** 10.1371/journal.pone.0169964

**Published:** 2017-01-27

**Authors:** Alice C. O’Farrell, Rhys Evans, Johanna M. U. Silvola, Ian S. Miller, Emer Conroy, Suzanne Hector, Maurice Cary, David W. Murray, Monika A. Jarzabek, Ashwini Maratha, Marina Alamanou, Girish Mallya Udupi, Liam Shiels, Celine Pallaud, Antti Saraste, Heidi Liljenbäck, Matti Jauhiainen, Vesa Oikonen, Axel Ducret, Paul Cutler, Fionnuala M. McAuliffe, Jacques A. Rousseau, Roger Lecomte, Suzanne Gascon, Zoltan Arany, Bonnie Ky, Thomas Force, Juhani Knuuti, William M. Gallagher, Anne Roivainen, Annette T. Byrne

**Affiliations:** 1 Department of Physiology and Medical Physics, Royal College of Surgeons in Ireland, Dublin, Ireland; 2 Turku PET Centre, Turku University Hospital and Åbo Akademi University, Turku, Finland; 3 UCD School of Biomolecular and Biomedical Science, UCD Conway Institute, University College Dublin, Belfield, Dublin, Ireland; 4 Roche Innovation Center Basel, F Hoffman La Roche, Basel, Switzerland; 5 Pathology Experts GmbH, Basel, Switzerland; 6 Oncomark Ltd, Dublin, Ireland; 7 Heart Center, Turku University Hospital and Åbo Akademi University, Turku, Finland; 8 Public Health Genomics Unit, National Institute for Health and Welfare, Helsinki, Finland; 9 UCD Obstetrics & Gynaecology, School of Medicine, University College, Dublin, National Maternity Hospital, Dublin, Ireland; 10 Université de Sherbrooke, Québec, Canada; 11 Department of Medicine, Hospital of the University of Pennsylvania, Philadelphia, United States of America; 12 Vanderbilt University School of Medicine, Nashville, United States of America; 13 Turku Center for Disease Modeling, University of Turku, Turku, Finland; University of Illinois at Chicago, UNITED STATES

## Abstract

Sunitinib is a tyrosine kinase inhibitor approved for the treatment of multiple solid tumors. However, cardiotoxicity is of increasing concern, with a need to develop rational mechanism driven approaches for the early detection of cardiac dysfunction. We sought to interrogate changes in cardiac energy substrate usage during sunitinib treatment, hypothesising that these changes could represent a strategy for the early detection of cardiotoxicity. Balb/CJ mice or Sprague-Dawley rats were treated orally for 4 weeks with 40 or 20 mg/kg/day sunitinib. Cardiac positron emission tomography (PET) was implemented to investigate alterations in myocardial glucose and oxidative metabolism. Following treatment, blood pressure increased, and left ventricular ejection fraction decreased. Cardiac [^18^F]-fluorodeoxyglucose (FDG)-PET revealed increased glucose uptake after 48 hours. [^11^C]Acetate-PET showed decreased myocardial perfusion following treatment. Electron microscopy revealed significant lipid accumulation in the myocardium. Proteomic analyses indicated that oxidative metabolism, fatty acid β-oxidation and mitochondrial dysfunction were among the top myocardial signalling pathways perturbed. Sunitinib treatment results in an increased reliance on glycolysis, increased myocardial lipid deposition and perturbed mitochondrial function, indicative of a fundamental energy crisis resulting in compromised myocardial energy metabolism and function. Our findings suggest that a cardiac PET strategy may represent a rational approach to non-invasively monitor metabolic pathway remodeling following sunitinib treatment.

## Introduction

Sunitinib Malate (Sutent^®^) is a small molecule tyrosine kinase inhibitor (TKI) clinically approved to treat gastrointestinal stromal tumors, metastatic renal cell carcinoma and pancreatic neuroendocrine cancers [1] and is currently being implemented in over 130 trials across diverse cancer indications [2]. Sunitinib competes with adenosine triphosphate (ATP) binding on several tyrosine kinases, including vascular endothelial growth factor (VEGF), platelet derived growth factor (PDGF), Fms-like tyrosine receptor kinase-3 and adenosine monophosphate [AMP]-activated protein kinase (AMPK) amongst others [3, 4]. Thus, sunitinib is thought to exert multiple effects on tumor growth, survival and angiogenesis. Despite widespread clinical approval, a significant toxicity profile has been reported [5]. Moreover, it is strongly contended that there has been a significant and widespread under-recognition of sunitinib cardiotoxicity [6]. Sustained hypertension and deterioration in left ventricular ejection fraction (LVEF) are the most commonly reported cardiotoxicities, with congestive heart failure (CHF) also observed [7–9]. The long term impact of sunitinib treatment on cardiovascular function is still largely unknown, with no widespread structured protocols, guidelines or follow-up programs focusing on cardiovascular care and survivorship-related issues in place [10].

While the precise mechanisms underlying sunitinib-induced cardiotoxicity require full elucidation, several causative factors have been implicated, particularly the role of AMPK inhibition and subsequent perturbations in cardiomyocyte energy metabolism. Sunitinib attaches to the ATP binding pocket of receptor tyrosine kinases acting as a competitive inhibitor of ATP, thus preventing activation and downstream signalling [8]. It has thus been shown to alter energy homeostasis in cardiomyocytes, *via* inhibition of AMPK leading to energy-conserving mechanisms due to stress conditions, with resultant defects in metabolism [4, 9, 11]. Nevertheless, it is likely (due to lack of drug specificity) that inhibition of other kinases may also be involved in the development of cardiotoxicity [4, 12]. Sunitinib-mediated mitochondrial dysfunction leading to cardiotoxicity, as well as decreases in ATP production have also been shown [7, 11, 13], ultimately suggesting that sunitinib exerts its cardiotoxic effects via perturbed kinase signalling related to energy metabolism.

Considering the impact of sunitinib on cardiomyocyte metabolism, we hypothesized that plasticity in substrate usage could represent a novel marker of sunitinib cardiotoxicity. The heart relies heavily on aerobic metabolism [14] and normally derives 60–70% of its energy from β-oxidation of long chain fatty acids, with the remainder derived from carbohydrate sources [15]. However, the heart is capable of re-modelling metabolic pathways as a result of chronic pathophysiological conditions [16].

We sought to i) assess the utility of PET tracers to detect early pathologic changes in cardiac metabolism, a hypothesis previously proposed [17, 18], and ii) investigate whether metabolic-PET could inform proteomic mechanistic studies relating to sunitinib induced cardiotoxicity. Glucose metabolism was monitored using [^18^F]-fluorodeoxyglucose ([^18^F]FDG) whilst [^11^C]acetate was used to monitor oxidative metabolism and myocardial perfusion. Mechanistically driven proteomic, immunohistochemistry and electron microscopy (EM) analyses were further implemented to unravel mechanistic aspects of the associated cardiotoxicity phenotype.

## Materials and Methods

### Animals

Female Balb/CJ mice (n = 36, 6–8 weeks, Charles River Laboratories, Sandwich, UK) and Sprague-Dawley rats (n = 12, 8 weeks, Harlan, Horst, The Netherlands) were housed in groups of 3–5, maintained on a 12 hour light/dark cycle, with free access to standard rodent chow and water. Animal experiments conformed to guidelines from Directive 2010/63/EU of the European Parliament on the protection of animals used for scientific purposes. Experiments were licensed and approved by the National Animal Experiment Board/Regional State Administrative Agency for Southern Finland (License: 4835/04.10.03/2011) *or* the Department of Health and Children, Dublin, Ireland (License: B100/3654). Protocols were reviewed by University College Dublin Animal Research Ethics Committee.

Animals were randomised and treated daily (Monday-Friday, 4 weeks, oral gavage) with 40 (mice) or 20 (rats) mg/kg sunitinib (Sequoia Research Products, Pangbourne, UK) reconstituted in sterile water. Vehicle control cohorts received sterile water. After 4 weeks animals were humanely euthanized using CO_2_ overdose followed by cervical dislocation.

### Physiological Measurements

Blood pressure and cardiac physiology parameters were assessed prior to and during treatment. Blood pressure was measured using the tail cuff method, whilst echocardiography was performed using the Vevo 770^®^ or 1200^®^ ultrasound scanner in mice and rats respectively.

### Positron Emission Tomography

The myocardial metabolic rate of glucose (MMRG) was assessed using [^18^F]FDG in mice. [^11^C]Acetate-PET was used to determine myocardial perfusion and oxidative metabolism in rats as previously described [19]. Pre- and “on-treatment” scans were performed in both models. [^18^F]FDG-PET scans were performed at University College Dublin, Pre-Clinical Imaging Core where cardiac imaging protocols for mice have been validated. [^11^C]Acetate imaging was performed at Turku PET Centre, Finland. Rats were used for [^11^C]acetate imaging as their larger size improves image resolution and tracer modelling. Cardiac imaging protocols using [^11^C]acetate have been established and robustly validated in rats at the Turku PET centre.

### Histopathology and Electron Microscopy Analyses

After 4 weeks hearts were collected for downstream analyses including histopathology and EM. Haematoxylin and Eosin staining was performed using standard protocols. Masson’s Trichrome staining was used to assess fibrosis, whilst apoptosis and myocardial microvessel density were assessed using terminal deoxynucleotide transferase (TdT)-mediated dUTP nick-end labelling (TUNEL) and anti-CD31 staining on formalin-fixed paraffin-embedded tissue sections. Lipid droplet accumulation was assessed both by EM and Oil Red O (ORO) staining.

### Proteomic Analyses

Liquid chromatography tandem mass spectrometry (LC-MS/MS) was implemented to interrogate the effect of sunitinib on myocardial signalling pathways after 4 weeks of treatment in the mouse model. Enzyme-linked immunosorbent assay (ELISA) was used to detect Mitochondrial Complex I in post-treatment myocardial lysates. Western blot analysis was used to confirm proteomic findings (succinate dehydrogenase complex, subunit A (SDHA) and hydroxyacyl-Coenzyme A dehydrogenase/3-ketoacyl-coenzyme A thiolase/enoyl-coenzyme A hydratase (trifunctional protein), alpha subunit (HADHA)) and to determine levels of phosphorylated-Acetyl co-enzyme A carboxylase (phospho-ACC), CD36 and hypoxia-inducible factor 1 alpha (HIF-1α).

Please refer to S1 File for full protocols.

### Statistical Analysis

For all experiments (except LC-MS/MS) a p-value of < 0.05 was deemed to be significant. Data were analysed between or within groups with unpaired / paired tests as described further in S1 File. Error bars are SEM unless stated otherwise. For LC-MS/MS analyses please refer to detailed methods in S1 File.

## Results

### Sunitinib Compromises Cardiac Function in Two Independent Rodent Models

Rodents were treated with the minimal dose at which robust anti-tumor efficacy has been observed across a range of rodent xenograft models [3] taking into account human plasma concentrations, drug-drug interactions and the prevalence of cardiac toxicities in the clinic [20].

**Fig 1:**
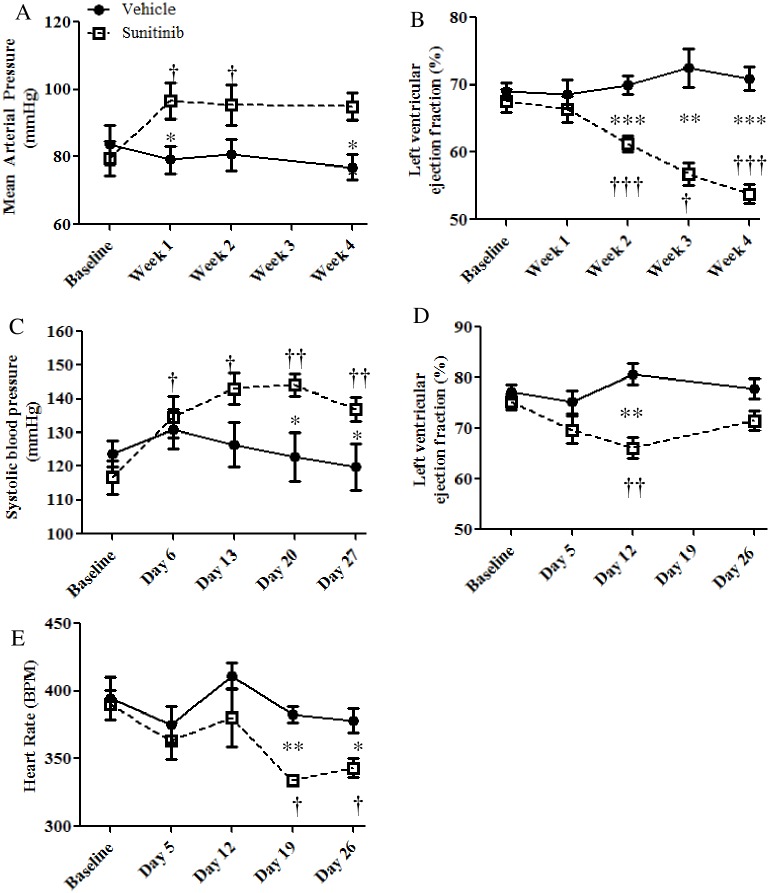
Physiological effects of sunitinib in two rodent models. Effects of sunitinib treatment on MAP (A, n = 9/group) and LVEF (B, n = 13/group) in female Balb/CJ mice. D-E) Effect of sunitinib treatment on systolic blood pressure (C), LVEF (D) and heart rate (E) in female Sprague-Dawley rats (n = 6/group, Significant difference between groups (unpaired t-test, *p<0.05 **p<0.01 ***p<0.001), Significant change from pre-treatment values (paired t-test, †p<0.05 ††p<0.01 †††p<0.001). Error bars = SEM)

To confirm that sunitinib caused clinically relevant changes in cardiovascular function of rodents, blood pressure and LVEF were monitored. In sunitinib treated mice, mean arterial pressure (MAP) increased significantly within the first week of treatment from 79.5 mmHg to 96.6 mmHg (Fig 1A), remaining elevated throughout the study period whilst LVEF (Fig 1B) fell significantly from an average of 68% (pre-treatment) to 61% (week 2) and 54% (week 4). LVEF in all treated animals exhibited a decrease compared to pre-treatment. Similar results were observed in a rat model where systolic blood pressure rose significantly from an average of 117 mmHg to 135 mmHg within the first 5 days of sunitinib treatment (Fig 1C). All sunitinib treated rats displayed an increase in systolic blood pressure. LVEF also fell in this model (Fig 1D), reaching significance at day 12. Some recovery was seen in treated animals towards the end of the treatment period, representing adaptation that has been observed elsewhere in a similar model [21]. Fractional shortening (S1A Fig), cardiac output (CO; S1B Fig) and heart rate (Fig 1E) were additionally measured in the rat model, all showing significant decreases during the treatment period.

### Sunitinib Alters Cardiac Metabolism as Measured by Metabolic PET

PET was used to non-invasively assess the impact of sunitinib on myocardial metabolism. In mice, PET was performed using [^18^F]FDG, a glucose analogue, to measure MMRG. In rats K_mono_ values, calculated from [^11^C]acetate-PET scans, were used to measure oxidative metabolism.

**Fig 2:**
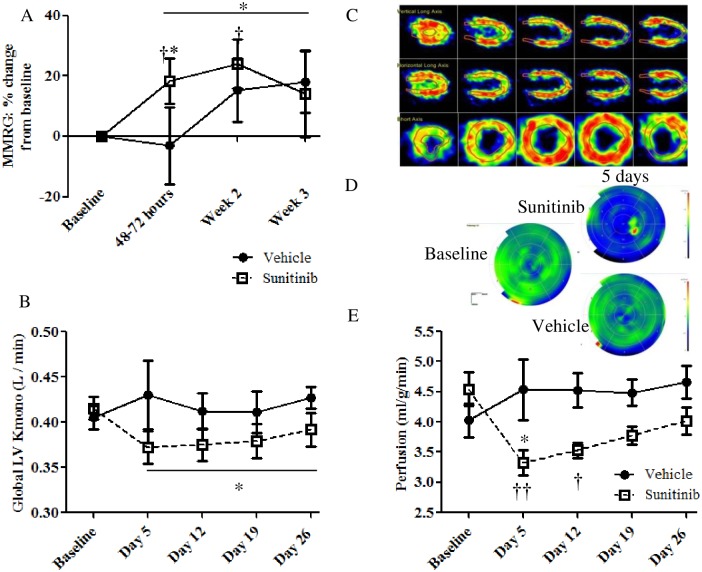
Effect of sunitinib treatment on myocardial metabolism and perfusion measured by PET. A) Myocardial metabolic rate of glucose (MMRG) (mice, n = 10/group), B) K_mono_ (rats, n = 6/group). C-E) The effects of sunitinib treatment on overall perfusion of the myocardium in the rat model: (C) Representative images of the myocardium as seen in Carimas software (version 2.7) which is used to select region of interest (ROI), subsequently used to construct 17 segment heat maps for the 11C-Acetate data (rats) (D) which are used to determine global perfusion values. D) Representative pre- and 5 days post-treatment in one treated and one vehicle rat E) The effects of sunitinib treatment on overall perfusion of the myocardium (rats, n = 6/group, * = significant difference between groups (unpaired t-test, p<0.05), Significant change from pre-treatment (paired t-test, †p<0.05 ††p<0.01), bar* indicates significantly different overall on-treatment values (two-way ANOVA p < 0.05) Error bars = SEM).

MMRG in mice increased significantly 48–72 hours following commencement of treatment, further increasing to 30% during the second week of treatment, before declining (Fig 2A). MMRG was significantly elevated in the sunitinib versus the vehicle group during the treatment period. MMRG variability may be explained by the requirement to conduct rodent cardiac PET studies in the non-fasted state to ensure generation of a detectable myocardial MMRG signal [22]. Pilot studies (data not shown) indicated that in the fasting state [^18^F]FDG update was largely below the limit of detection. While this represents a technical limitation in rodent imaging, clinical cardiac [^18^F]FDG-PET implements robust protocols to control patient glucose levels.

K_mono_ values (measured using [^11^C]acetate-PET) were significantly lower in the sunitinib treated animals during the treatment period (Fig 2B), indicating a sustained decrement in myocardial oxygen consumption.

### Sunitinib Treatment Induces a Significant Decrease in Myocardial Perfusion

[^11^C]Acetate-PET was also used to determine myocardial perfusion in rats (Fig 2C–2E). Following 5 days of treatment, myocardial perfusion had decreased significantly compared to pre-treatment values, by an average of 38% (Fig 2E). Decreases of up to 80% in individual animals were seen. Starting at day 12, some recovery was seen in treated animals, although perfusion never returned to pre-treatment values.

### Changes in Myocardial Structure, Apoptosis, Microvessel Density or Fibrosis, are not Observed in Sunitinib Treated Animals

**Fig 3:**
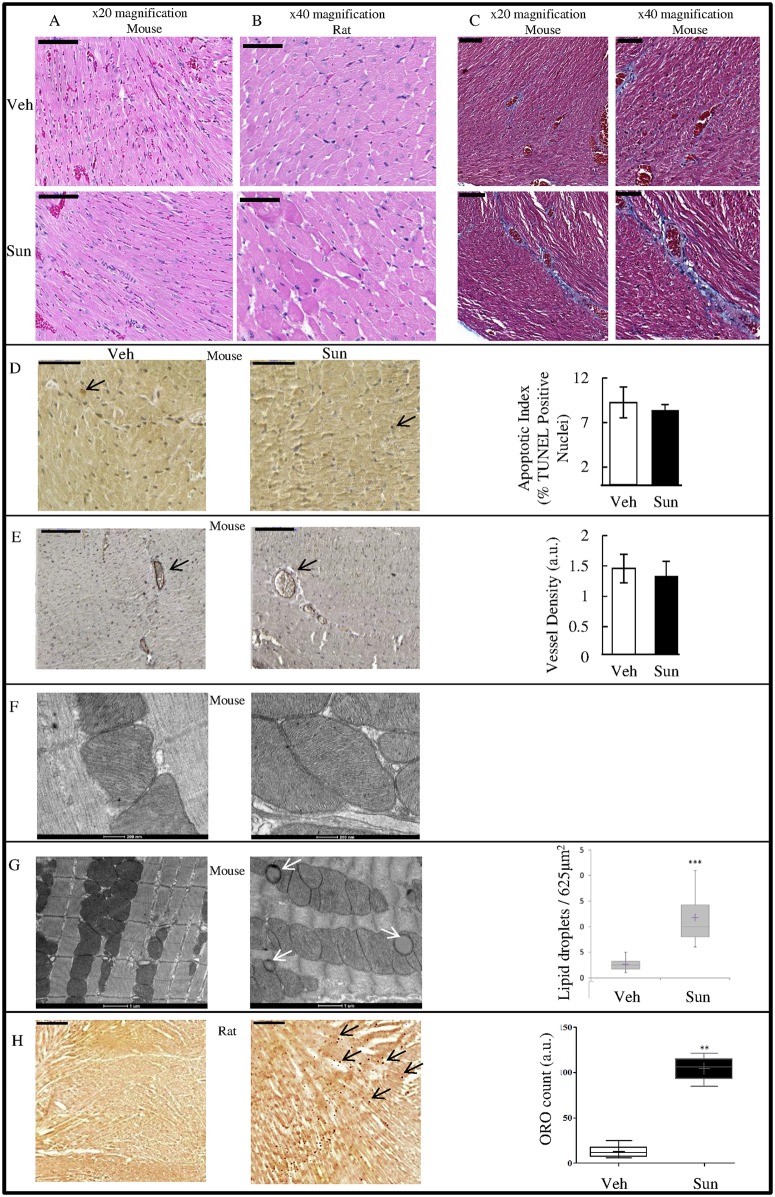
Histopathologic assessment of rodent myocardium following sunitinib treatment. All error bars represent SEM unless otherwise indicated. H&E staining in mice (A) and rats (B) Magnification as indicated, scale bars (black) 100 μM and 50 μM respectively. C) Masson’s trichrome staining to assess fibrosis in the myocardium of vehicle versus sunitinib treated mice (scale bars (black) represent 100 μm (left) and 50 μm (right), magnification as indicated). D) TUNEL staining for the assessment of apoptosis in mice (40 × magnification; scale bar (black): 50μm. 1000 nuclei were counted per organ using ImageJ software. % TUNEL positive nuclei shown). E) Microvessel density in mice (CD31 immuno-staining). Representative images shown; scale bar (black) 50 μm, x 40 magnification. Vascular density is expressed as % CD31 positive pixels to total pixels captured in each image. F and G) EM analysis of cardiac tissue (mice) to determine the presence of mitochondrial damage [high magnification; x 29000] (F) and the presence of lipid droplets (white arrows) [lower magnification; ×11500] (G), scale bars as indicated ** p = <0.01, n = 4/group. H) ORO analysis in rat heart cryosections to determine lipid droplet accumulation (black arrows indicate representative red lipid droplets, scale bars represent 500 μm; magnification is ×5; n = 3/group. Quantification a.u, arbitrary units **p < 0.01, compared with vehicle. Values are means ± SD).

Following 4 weeks of treatment all animals were humanely euthanized and hearts collected for post-mortem analyses. Myocardial structure, including hypertrophy, fibrosis, apoptosis and microvessel density were assessed. Sunitinib did not affect morphology of the myocardium in either mice or rats (Fig 3A and 3B). In mice, overt pathologic cardiac fibrosis was not observed, whilst TUNEL staining indicated no significant difference in levels of apoptosis (Fig 3C and 3D respectively). Microvessel density remained unchanged (Fig 3E and S2 Fig). EM analysis indicated no effect on mitochondrial ultrastructure (Fig 3F).

### Sunitinib Leads to Increased Presence of Lipid Droplets in the Myocardium

EM analysis of mouse myocardium revealed a significantly higher (4.6-fold) number of lipid droplets, clustering adjacent to the mitochondria in sunitinib treated animals (Fig 3G). ORO staining confirmed this finding in rat myocardium, where the presence of neutral triglycerides and lipids was approximately 8-fold higher in sunitinib treated animals (Fig 3H).

### Sunitinib Treatment Perturbs Key Pathways Involved in Energy Balance in the Myocardial Metabolic Proteome

To interrogate the impact of sunitinib treatment on an extended dynamic range of proteins, LC-MS/MS and IPA^®^ (QIAGEN Redwood City, www.qiagen.com/ingenuity) was performed on murine myocardial tissue extracts (harvested after 4 weeks of treatment) to identify pathophysiological pathways significantly enriched following sunitinib treatment (See S3 and S4 Figs and S1 and S2 Tables for a full list of identified proteins, hierarchical clustering and PCA).

**Fig 4:**
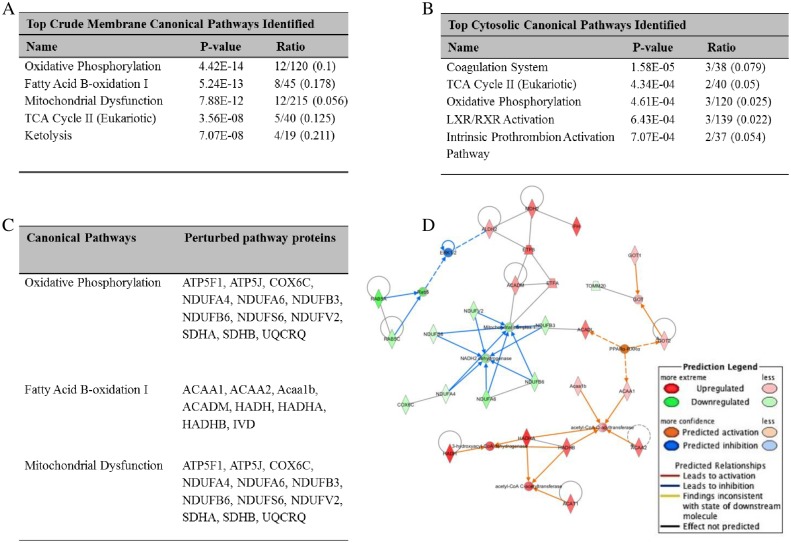
Proteomic analyses of sunitinib effects in myocardial tissue. The most significantly perturbed canonical pathways from (A) crude membrane and (B) cytosolic fractions from sunitinib treated and control mouse myocardial tissue (n = 4/group) C) Proteins perturbed in the three most significantly enriched canonical pathways. D) Ingenuity Pathway Analysis (IPA) to determine the most significantly enriched network which is centred on Mitochondrial complex 1. Colour intensity indicates the degree of up- (red) or down- (green) regulation. IPA predictions are shown in blue (predicted inhibition) and orange (predicted activation). Black indicates no predicted effect. Continuous lines indicate a direct relationship between two proteins; a discontinuous line indicates indirect association.

The top three canonical pathways perturbed were oxidative phosphorylation (p = 4.42E-14), fatty acid β-oxidation I (p = 5.24E-13) and mitochondrial dysfunction (p = 7.88E-12). These, along with other significantly enriched canonical pathways are shown in Fig 4A and 4B. The number of identified proteins involved in each pathway is indicated by the “ratio” column and identified proteins within the “top pathways” are listed in Fig 4C.

Significant regulatory networks identified by IPA to be affected by sunitinib were also examined (those with a score > 20 and > 10 focus molecules), the most significant network (score = 60 and focus molecules = 26) being centred on Mitochondrial Complex 1 (Fig 4D). MS proteomic data have been deposited to the ProteomeXchange Consortium [23] via the PRIDE partner repository [24] with dataset identifier PXD001888.

### Validation and Confirmation of Key Proteomic Findings

**Fig 5:**
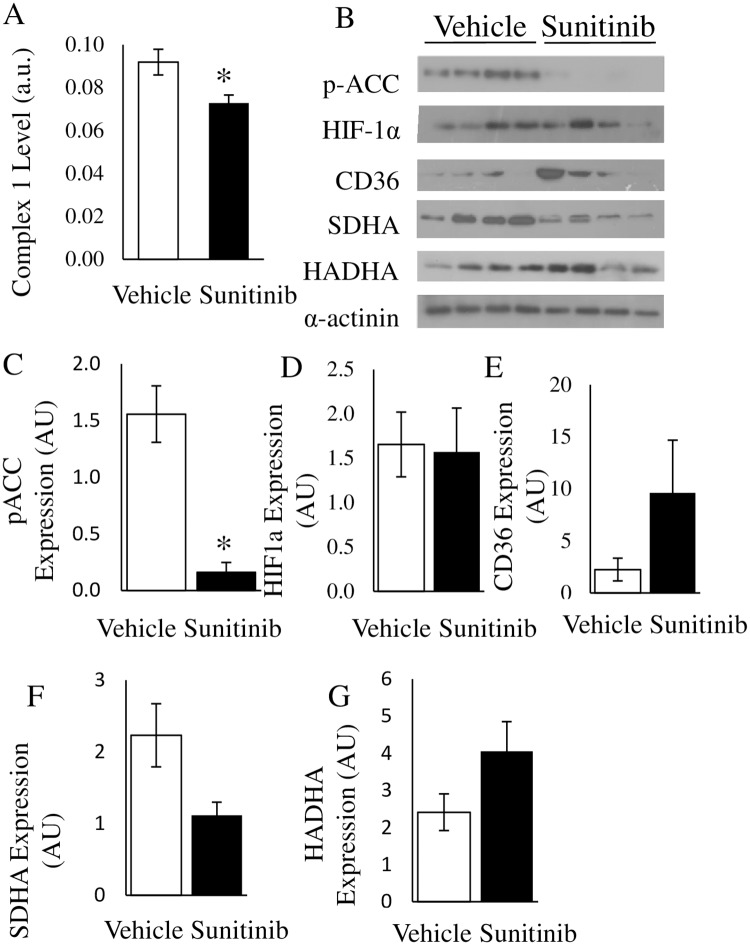
Confirmation of proteomic findings in mouse myocardial tissue. A) ELISA to measure Mitochondrial complex 1 levels in myocardial tissue lysates. B) Immunoblot analysis of lysates from the cytosolic fraction of myocardial tissue from vehicle or sunitinib treated animals (n = 4/group) as described. C-G) Densitometry assessment of the presented western blots as described (* p < 0.05, error bars represent SEM).

Key sunitinib pathway effectors implicated by LC-MS/MS were confirmed by ELISA and Western blot. A decrease in Mitochondrial Complex I (ELISA) (Fig 5A), in the hearts of treated mice supported proteomic findings, implicating complex down-regulation as central to the most significantly enriched network identified by IPA (Fig 4D). SDHA, a subunit of Complex II, was decreased in myocardial tissue following treatment (S2 Table, Log^2^ relative rank = -0.684); Western blot analysis showed a 50% reduction compared to vehicle (Fig 5B and 5F). HADHA, a protein that mediates the mitochondrial fatty acid beta-oxidation pathway, appeared to be perturbed in the cytosolic myocardial fraction of tissues of sunitinib treated mice (S2 Table, Log^2^ relative rank = -1.069). However this observation was not confirmed via Western blot (Fig 5B and 5G).

To determine whether sunitinib treatment resulted in inhibition of AMPK (mouse model) phospho-ACC, a known substrate of AMPK [11], was assessed. A 90% reduction (p < 0.05) was observed in the hearts of sunitinib treated animals compared to vehicle (Fig 5B and 5C). Due to the increase in myocardial lipid droplet accumulation, CD36, a protein required for lipid delivery into cardiomyocytes, was assessed. We observed a 4-fold increase in the levels of CD36 in the cardiac tissue of treated mice compared to controls (Fig 5B and 5E). Sunitinib treatment did not impact HIF-1α (a marker of hypoxia) protein levels (Fig 5B and 5D).

## Discussion

The burgeoning field of cardio-oncology is driven by cardiovascular complications that occur as a direct result of cancer treatments. It is widely accepted that early recognition of cardiovascular side effects is vital to allow long-term continuous therapy [25]. Considering the impact of sunitinib on cardiac energy balance, we hypothesized that plasticity in cardiac energy metabolism could represent an early marker of cardiotoxicity. Cardiac PET was implemented to investigate alterations in myocardial glucose and oxidative metabolism. We further implemented robust and mechanistically driven proteomic and histochemical analyses to interrogate the effects of sunitinib on metabolic pathways and to further uncover aspects of the sunitinib induced cardiotoxicity phenotype.

In both rat and mouse models, sunitinib treatment lead to the development of hypertension and also to decreased LVEF, mirroring the effects of sunitinib seen in the clinic. PET during the treatment period indicated early alterations in metabolism; increased glucose uptake and metabolism (MMRG) in mice and decreased oxidative metabolism (K_mono_) in rats, coupled with decreased perfusion in the myocardium as early as 5 days following commencement of treatment. Despite these apparent metabolic changes no differences were seen in the morphology of the myocardium in treated animals (mice and rats) after 4 weeks of sunitinib treatment. Nevertheless, lipid droplet accumulation was evident in both models after 4 weeks of sunitinib treatment. LC-MS/MS data in mice corroborated the imaging findings insofar as metabolic pathway markers appeared as those most significantly perturbed following sunitinib treatment.

A limited number of rodent studies have reported perturbations in discrete cardiovascular parameters following sunitinib treatment [20, 21, 26, 27]. A recent study suggested that left-ventricular fractional shortening is significantly decreased in mice that had received 28 days of sunitinib treatment [27]. However, to the best of our knowledge our study is the first demonstration of two concurrent ‘non-interventional’ rodent models which present with a clinically relevant and *early* cardio-toxicity phenotype following sunitinib treatment in the absence of non-physiologic perturbations such as transaortic constriction or chemical induction [7, 20]. Balb/CJ mouse and Sprague-Dawley rat models presented with increased blood pressure accompanied by decreased LVEF (Fig 1). LVEF recovery was observed in the rat model after 3 weeks of treatment. Adaptation to sunitinib cardiovascular insult has been previously noted in rat models [21]. Nevertheless, basic cardiac histology remained unchanged following sunitinib treatment in both models, with no evidence of fibrosis or hypertrophy (Fig 3A–3C). These data concur with previous studies [20, 21], although others have shown LV fibrosis in female mice following sunitinib treatment [27]. As previously observed *in vivo* [7, 13] and *in vitro* [28] no change in the incidence of apoptosis was evident in the myocardial tissue of sunitinib treated animals (Fig 3D).

Implementing our mouse model, we observed that glucose uptake and metabolism, quantified by MMRG, increased after 48 hours of sunitinib treatment (earlier than any changes in LVEF, which occurred after 2 weeks), suggesting a rapid shift in the balance of metabolites available to, or being used by, cardiomyocytes. For example, a shift from fatty acid metabolism to glucose improves oxygen efficiency for ATP synthesis [16]. As mentioned above, in the mouse model, MAP increases and remains elevated whereby LVEF shows a continuous decline. In parallel, MMRG is elevated above baseline for the first two weeks of treatment before returning (almost) to pre-treatment values by Week 3. We hypothesise that early changes in MMRG reflect metabolic substrate plasticity, whereby glucose metabolism increases in response to stress (as seen in other disease states e.g. chronic hypertension [16]). Nevertheless, as the fate of the tracer is ultimately unknown, we cannot rule out glycogen accumulation as underpinning detectable MMRG increase [29]. Increased glycogen has been shown to correlate with a relative increase in [^18^F]FDG uptake following ischemic injury in swine hearts, suggesting that increased rates of glucose transport and/or phosphorylation are important determinants of relative accumulation of glycogen [30]. Working heart studies using radiolabelled isotopes in isolated hearts could be used to further interrogate these findings. Although not significantly different from baseline elevated MMRG in vehicle treated animals was also observed which may reflect variations in glucose levels at time of imaging. *Ex vivo* measurements of myocardial glucose uptake at each time point could provide further insight. By Week 4 of treatment, while LVEF decline persists (indicating non-adaptation in the mouse model) our data suggests that the myocardium has attempted to revert to fatty acid metabolism; histological analyses revealed the presence of cardiac lipid droplets (Fig 3F and 3G). While lipid droplet accumulation is a phenomenon previously observed *in vitro* [28], to the best of our knowledge, this is the first observation of cardiac lipid droplet accumulation following sunitinib treatment *in vivo*. However, over-accumulation of lipids in the myocardium has been shown to correlate with cardiac dysfunction (“lipotoxic cardiomyopathy” [16, 31]) resulting in decreased fatty acid oxidation, accumulation of toxic lipid intermediates, and contractile failure. This would explain the persistent reduced LVEF still observed at Week 4. At least three mechanisms may be considered: 1) reduced fatty acid oxidation and oxidative metabolism, as supported by proteomic data (discussed below); 2) reduced phospho-ACC (Fig 5B and 5C) which may contribute to the build-up of lipid deposits by increasing lipid synthesis and inhibiting fatty acid oxidation; 3) increased presence of fatty acid transporter proteins (e.g. CD36) (Fig 5B and 5E). Further studies are required to fully elucidate these pathways. Notwithstanding that MMRG data is unavailable for the rat model, we nevertheless still observe enhanced accumulation of lipid droplets in the rat heart (Fig 3H) even though LVEF largely returns to normal by Day 26 in this model. These data suggest individual (or in this case, inter-species) variability in the adaptation process, whereby despite perturbed cardiac metabolism, some hearts retain the ability to adapt to sunitinib treatment. Further worked is warranted to interrogate individual adaptation response.

It has previously been shown *in vitro* [32] and in recently published *in vivo* data that treatment with sunitinib leads to myocardial oxidative stress [33]. Thus [^11^C]acetate-PET was performed in rats to determine whether this tracer could detect early changes in myocardial oxygen consumption. As acetate is fully metabolised in the TCA cycle and due to tight coupling between the TCA cycle and oxidative phosphorylation, early clearance rate of [^11^C]acetate after intravenous administration correlates strongly with myocardial oxygen consumption [34]. Significantly reduced oxidative metabolism was observed in the sunitinib group over the treatment period (Fig 2B).

The initial uptake of the [^11^C]acetate tracer is indicative of myocardial perfusion. Our results indicate that treatment with sunitinib leads to a significant reduction in perfusion after just 5 days of treatment, with a gradual recovery over the treatment period (Fig 2E). Myocardial ischemia as a result of decreased perfusion (which has been reported in patients following sunitinib treatment [35]) could also lead to perturbation of fatty acid oxidation and an accumulation of lipids, with glucose becoming the primary substrate for both increased anaerobic glycolysis and for continued (although diminished) oxidative metabolism. This metabolic switch may be a prerequisite for continued energy production and cell survival [29]. As such, our [^11^C]acetate data could suggest a reduction in metabolic rate and shift towards anaerobic metabolism manifesting soon after commencement of sunitinib treatment. Further work is required to assess this hypothesis. Interestingly, both myocardial ischemia and changes in cardiac metabolism have been implicated in the appearance of clinical arrhythmias [35, 36]. Thus, it is noteworthy that bradycardia was observed in the current study (Fig 1E). Sunitinib can lead to a prolongation of the QT interval [37], which is often associated with low heart rates [38]. This has previously been observed both *in vitro* and in preclinical and clinical settings [26, 28, 39] although mechanisms are yet to be fully elucidated.

The perfusion imaging results observed in rats indicated that following the initial decrease in perfusion, there was some recovery over the course of the treatment period. The effects of sunitinib on microvessel density (using the CD31 marker) and myocardial hypoxia (using HIF1α) in the mouse model were thus also investigated and no differences between vehicle and sunitinib groups were apparent after 4 weeks of treatment. N.B. As the [^11^C]acetate-PET perfusion data indicates recovery by 4 weeks this is not unexpected. Interestingly, up-regulated PHD3 (Prolyl hydroxlase 3; a controller of HIF) expression has been observed in mouse myocardial tissue following 7 days of sunitinib treatment [20], which supports our early observations, where perfusion was at its lowest. Elsewhere, up-regulation of HIF-1α gene expression was observed following 21 days of sunitinib treatment, although Western blot analysis showed a non-significant increase of HIF1α in the nuclear content [40]. It has been suggested that cardiovascular pericytes are a primary cellular target of sunitinib-mediated cardiotoxicity. In the myocardium of sunitinib treated mice, decreases in PDGFR-β (pericyte marker) expression in cardiac lysates, decreased co-localisation of pericytes with CD31 and concurrent coronary microvessel tortuosity and vascular permeability have been reported [20]. The initial decrease in perfusion in the rat model may therefore be indicative of the effect of sunitinib on pericytes (through its known interaction with PDGF). Nevertheless, at 4 weeks, there was no difference between CD31 expression between groups (Fig 3E) and perfusion had recovered almost to baseline further indicating coronary vasculature recovery in these models. Finally, it must also be considered that myocardial blood flow may be affected by overall cardiac performance. Cardiac output (CO) measurements indicated a significant albeit modest decrease following 6 days of treatment (S1B Fig) in rats. Isolated heart studies combined with *ex vivo* analysis of treated animals could confirm whether the decrease in perfusion reflects a reduction in cardiac output, a compensatory effect due to reduced myocardial oxygen demand, or a direct effect on coronary microvascular function. The long term effects of early changes in perfusion are currently unknown.

It has previously been reported that sunitinib may cause overt mitochondrial damage in rodents and human cardiomyocytes [7, 11, 13]. In the current study, EM analysis indicated no effect on mitochondrial ultrastructure (Fig 3F). It is unclear why this effect is not evident in our models, however sex and mouse strain differences may be relevant here [27]. Sunitinib induced decline in LVEF has been shown to be reversible in humans [41]. It is possible that recovery may occur if mitochondrial structural integrity can be preserved. In our study, mice that develop LVEF dysfunction, but maintain mitochondrial integrity may represent those patients where treatment withdrawal would allow LVEF recovery. It will be important in the future to confirm whether mitochondrial dysfunction is a primary or a secondary event in the sunitinib induced cardiotoxicity sequela.

LC-MS/MS pathway analysis of myocardial tissue was performed to further provide mechanistic insight into the sunitinib-induced cardiotoxic phenotype. Top canonical pathways perturbed were those involved in oxidative phosphorylation, fatty acid β-oxidation I, mitochondrial dysfunction and TCA Cycle II (Fig 4C). Additionally, IPA (confirmed by ELISA, Fig 5A) indicated that the most significant network among those affected by sunitinib was centred on Mitochondrial Complex 1, which plays an essential role in oxidative phosphorylation and generation of cellular ATP [42]. LC-MS/MS findings were further validated using Western blot analysis: SDHA (a member of both the TCA cycle and oxidative phosphorylation pathways), was also down-regulated in myocardial tissue. As the heart relies on aerobic oxidative substrate metabolism for the generation of ATP any decline in mitochondrial activity may lead to perturbation of respiratory pathways, decreased ATP production and ultimately heart failure [43].

Future studies will include tissue collection at early time points to further inform mechanistic interrogation of early toxicity pathways to better corroborate imaging data and also to perform [^18^F]FDG-PET imaging in both species. Moreover, given the lack of histological findings, additional studies are required to clarify the relationship between hypertension, sunitinib, and cardiomyopathy. Specifically, hypertensive models (either spontaneously hypertensive animals or implementing interventional hypertensive agents) may elucidate whether sunitinib directly (via perturbation of tyrosine kinases) or indirectly (via induced acute hypertension) causes early increases in MMRG.

In the current study the mouse model was used for all mechanistic investigations to corroborate [^18^F]FDG-PET data—a protocol established and validated in mice at the University College Dublin Pre-clinical Imaging Core Facility Dublin. The rat model was subsequently implemented to determine whether [^11^C]acetate could be used to investigate additional effects of sunitinib (on perfusion and oxidative metabolism) and whether it would be suitable as an early imaging biomarker of toxicity. In this case PET protocols established and validated in rats at the PET Centre, Turku, Finland were exploited. Data from the rat model suggests a physiological adaption to prolonged sunitinib treatment following initial disruption (such adaption has been reported previously in rats [21]) manifesting as recovery of LVEF and perfusion over the course of the treatment period. Nevertheless, recovery in oxidative metabolism, blood pressure or heart rate is not observed and there is lipid droplet accumulation in the myocardium. There was no similar recovery in LVEF in the mouse model. These data may thus recapitulate individual (or in this case, species) variability in the adaptation process, whereby despite perturbed cardiac metabolism, some hearts retain the ability to adapt to sunitinib treatment. Further work is warranted to interrogate individual/ species specific adaptation response mechanisms.

Current ‘on treatment’ manufacturers’ guidelines mandate that oncology patients should be monitored by electrocardiography and assessed for CHF and hypertension. In the clinic LVEF assessment by echocardiography is the most common practice [44, 45]. Nevertheless, quantifiable reductions in LVEF may underestimate true cardiac damage, as myocardium compensatory reserve can allow for adequate ventricular output despite sub-clinical dysfunction. Data may be further confounded by inherent interpretation subjectivity [46, 47]. Thus, echocardiographic evaluation of changes in LVEF may not represent an ideal method for early evaluation of cardiotoxicities following cancer therapy as echocardiographic methods provide little insight into drug mechanism. Thus, an effective strategy for the early, mechanism driven detection of cardiac damage in the context of sunitinib treatment may be warranted. Our data indicates that sunitinib induces early changes in myocardial metabolism that precede the development of overt cardiotoxicity and supports the potential utility of PET to monitor substrate remodeling pathways. The plausibility of this method is supported by a 2011 case study, following the sudden death of a patient 28 months after commencing TKI combination therapy. The patient died from a sudden myocardial infarction with cardiogenic shock, but retrospective analysis of sequential [^18^F]FDG-PET scans were indicative of cardiac toxicity developing during the period of concomitant administration of TKIs [18]. Further clinical studies are warranted to fully understand the role of cardiac metabolic pathway remodeling and PET in the cardio-oncology setting, particularly relating to the early identification of cardiotoxicities and their reversibility. This is especially important when considering the expected increase in the application of TKIs. Understanding the perturbations that occur with sunitinib could help inform the design of personalized, targeted pharmacologic therapy. There is little guidance on the most efficacious pharmacologic therapy, given limited mechanistic understanding. Thus, if it is determined that there are major adverse changes in oxidative stress or cardiac metabolism, use of medications which improve these axes (e.g. beta blockers) may be the preferred cardioprotective strategies. This work could further inform the development of newer cardioprotective strategies that specifically target and improve metabolism.

## Supporting Information

S1 File Supplemental Methods. (PDF)

S1 Fig The effect of sunitinib on A) fractional shortening and B) cardiac output (CO) in rats. (PDF)

S2 Fig CD31 micro-vessel segmentation output. (PDF)

S3 Fig LC-MS/MS data analysis of insoluble and soluble fractions. (PDF)

S4 Fig Hierarchical clustering and principle component analysis. (PDF)

S1 Table Significantly changed proteins identified by LC-MS/MS in the crude membrane fraction. (PDF)

S2 Table Significantly changed proteins identified by LC-MS/MS in the cytosolic fraction. (PDF)
