# Supplementary material for: A Novel Positron Emission Tomography (PET) Approach to Monitor Cardiac Metabolic Pathway Remodeling in Response to Sunitinib Malate
Source: PLoS One. 2017 Jan 27;12(1):e0169964. doi: 10.1371/journal.pone.0169964 (PMC5271313; doi:10.1371/journal.pone.0169964)
Supplement: S1 File — (PDF) [file pone.0169964.s001.pdf]

# S1 File. Supplemental Methods

## Additional information for calculation of animal numbers

Initial group size for mice was based on the calculation $n = (Z_a + Z_b)^2 (2s^2 / d^2)$, where a two-tailed test, power of 80% and a p-value of <0.05 was deemed to be significant. Results from previous studies where treatment with a VEGF-targeting monoclonal antibody led to an increase in blood pressure of 8 mmHg (126 mmHg [treated] compared to 118 mmHg [control] with a standard deviation of 6 mmHg gave a group size of 9. For echocardiography group size was 13 as additional mice were required to supply tissue for proteomic analysis. Group size was dropped in rats to n = 6 based on the initial results seen in mice, where the difference in blood pressure at after a week of treatment were used to input data into the equation above. Individual mice underwent blood pressure monitoring + echocardiography, blood pressure monitoring + [$^{18}$F]FDG-PET or echocardiography + [$^{18}$F]FDG-PET. All rats were subject to blood pressure monitoring, echocardiography and [$^{11}$C]acetate-PET. Researchers were not blinded to conditions during imaging however IHC and LC-MS/MS studies were performed blinded, with the 2 groups coded prior to analysis.

## Blood pressure monitoring

Blood pressure was measured using the Visitech BP-2000 Blood Pressure Analysis System (Apex, NC, USA) and the tail cuff method. Mice were restrained, the tail fed through a cuff and held in place using surgical tape during analysis. Mean arterial pressure (MAP) was calculated using an average of at least 3 blood pressure recordings. Mice were conditioned prior to the experiment starting by running 4-6 blood pressure measurement sessions prior to baseline measurements being taken. Blood pressure measurements in rats were recorded using the tail-cuff method (NIBP System/ PowerLab, ADInstruments, Bella Vista, NSW, Australia). Animals were lightly anaesthetised (1.5% isoflurane/$O_2$) for this procedure. Average systolic blood pressure was determined over a period of at least 3 consecutive readings.

## Echocardiography

Ultrasound was used to assess cardiac function. Animals were anaesthetised using 2.5% isoflurane/$O_2$ and placed on a heated platform. Anaesthetic was maintained via a nose cone. In mice (Vevo 770, 704 40 MHz probe, Visual Sonics, Toronto, Canada) the parasternal short axis view was used to generate an M-mode image and LVEF was subsequently calculated using the integrated LVEF algorithm function. In rats (Vevo 2100, MS250 transducer, Visual Sonics, Toronto, Canada), the long axis view was used to generate an M-mode image. LVEF, fractional shortening (FS), cardiac output (CO) and heart rate (HR) were all calculated using this image.

## Positron Emission Tomography (PET)

[$^{18}$F]Fluorodeoxyglucose ([$^{18}$F]FDG)-PET cardiac imaging was performed using a dedicated small animal system (Triumph LabPET 4, Trifoil Imaging (formerly Gamma Medica), Chatsworth, CA, USA). Non-fasted animals were warmed in a heating chamber 20 minutes before being anaesthetised using 2.5% isoflurane/$O_2$. A tail vein catheter was inserted before transferring the anaesthetised animal onto a heated PET scanning bed. To assess myocardial glucose metabolism, a 46 minute scan was initiated 1 minute prior to an automated injection of $10 \pm 2$ MBq of [$^{18}$F]FDG in 80 μL of saline. Respiration, temperature and electrocardiography (ECG) were monitored for duration to ensure repeatability of physiological condition of animal on scanner. Blood glucose level was measured (Accu-chek, Aviva, Roche, Burgess Hill, UK) before and after scanning and average used for Patlak analysis. For analysis, list mode PET data was reconstructed using commercial software (LabPET version 1.12.1, General Electric, Bucks, UK) with a maximum likelihood expectation maximization (MLEM) algorithm set to 15 iterations into 29 frames of $1 \times 80$ seconds, $20 \times 10$ seconds, $2 \times 193$ seconds, and $6 \times 349$ seconds.  Time activity curves were obtained using PMOD software (version 3.2

PMOD Technologies Ltd, Zürich, Switzerland)) and defining regions of interest (ROI) on the myocardium, liver and left ventricle with at least two slices per ROI. Patlak analysis was performed as described previously [1] with a surrogate image-derived input function [2], which uses a hybrid of left ventricle and liver time-activity curves giving a relative measure of myocardial metabolic rate of glucose (MMRG).

Myocardial perfusion and oxygen consumption were assessed by [$^{11}$C]acetate-PET, using a dedicated small-animal PET scanner (Inveon Multimodality, Siemens Medical Solutions Knoxville, TN, USA). Rats were anaesthetised using 2.5% isoflurane/$O_2$. To assess the myocardial perfusion and oxygen consumption, a slow 11 ± 2 MBq bolus of [$^{11}$C]acetate was intravenously injected via a tail vein catheter and a 10 min dynamic PET acquisition was performed.

[$^{11}$C]Acetate PET data acquired in a list-mode was iteratively reconstructed with the ordered-subsets expectation maximization 2D algorithm (OSEM2D) into dynamic 5 × 2 s, 5 × 10 s, 4 × 30 and 7 × 60 frames. Quantitative image analysis was performed using Carimas 2.63 software (Turku PET Centre, Turku, Finland; http://www.turkupetcentre.fi/carimas/). Myocardial perfusion was determined using a single-compartment model and expressed as rate constant $K_1$ (mL/min). The values of the left ventricular myocardium were displayed as polar maps, which were normalised to own maximum. In order to study regional myocardial perfusion, the polar maps were also analysed using the American Heart Association 17 segment model. Myocardial oxygen consumption was assessed by applying monoexponential fitting to calculate [$^{11}$C]acetate clearance rate $K_{mono}$ (1/min).

# Tissue Preparation, Histological Examination and Microscopy

Samples for light microscopy were fixed in 4 % formaldehyde for 48 hours. Tissues were embedded in paraffin and 5 µm thick sections cut. Routine haematoxylin and eosin (H&E) staining was performed to facilitate histological evaluation by a veterinary pathologist.

Samples for transmission electron microscopy (TEM) were fixed in 2.5 % glutaraldehyde in 0.1 M Sørensen's phosphate buffer for a minimum of 2 hours at room temperature and post-fixed in 1% osmium tetroxide in Sørensen's phosphate buffer for 1 hour at room temperature. Subsequently, specimens were dehydrated then infiltrated and embedded in resin. Areas of interest were identified using 1 µm toluidine blue stained sections examined by light microscopy (Leica DMLB, Leica Microsystems, Wetzlar, Germany), and ultrathin (80 nm) sections were obtained using a Leica EM UC6 ultramicrotome (Leica Microsystems, Wetzlar, Germany). These sections were collected on 200 mesh thin bar copper grids, stained with uranyl acetate (20 min) and lead citrate (5 min) and examined by transmission electron microscopy (Tecnai G2 20 TWIN, FEI Company, Oregon, USA) using an accelerating voltage of 80 kV.

# TUNEL assay

Apoptosis was detected using a terminal deoxynucleotide transferase (TdT)-mediated dUTP nick-end labelling (TUNEL) assay. In brief, heart sections were subjected to biotin-streptavidin-horseradish peroxidase (HRP) labelling of DNA strand breaks using the Dead End Colorimetric Apoptosis Detection System (Promega, Southampton, UK). DNA breaks are subsequently detected using the HRP substrate peroxide and the chromogen diaminobenzidine (DAB). Sections were counterstained with hematoxylin. For each animal and therefore each organ, 1000 nuclei were counted using ImageJ software (National Institute of Health (NIH), Bethesda, MD, USA) and the level of apoptosis was expressed as % TUNEL positive nuclei.

# Oil Red O staining

To investigate the presence of neutral lipids in mouse myocardium, Oil red O (ORO) staining was performed. Stock solutions were prepared by adding 2.5 g of ORO (Sigma-Aldrich, St. Louis, MO, USA) to 400 mL of 99% (vol/vol) isopropyl alcohol and further dilutions performed $dH_2O$ to achieve a ratio of 6:4. The solution was filtered to yield a working solution. For staining, frozen rat hearts

were embedded in optimal cutting temperature (OCT) formulation (Fisher Scientific, Pittsburgh, PA, USA), and cryo-sections of 10 µm were cut and stained with ORO working solution for 15 min at RT. Sections were then rinsed in running tap water for 90 min before being mounted with a water-soluble mounting medium and set for 10 min at RT before being sealed. The slides were then scanned with a digital slide scanner (Pannoramic 250, 3DHISTECH Kft, Budapest, Hungary) and the areas positive for lipid staining quantified by ImageJ software (NIH). Graph Pad Prism version 5 software (GraphPad Software, Inc., La Jolla, CA, USA) was used for statistical analysis. All values are shown as mean ± standard deviation (SD). One-way analysis of variance (ANOVA) followed by the least significant difference calculation was used to analyse differences between groups. P values of less than 0.05 were considered to be statistically significant.

# Masson's Trichrome staining

To investigate the presence of fibrosis in the hearts of mice treated with sunitinib Masson's trichrome staining was performed. Briefly, heart slides were deparaffinised and rehydrated then immersed in preheated Bouin's Solution (Sigma HT101128) for 15 min at 56°C, washed in running tap water, stained in Wiegert's Iron Hematoxylin for 10 min, rinsed in deionized water, stained in Biebrich Scarlet-Acid Fucshin for 5 min, rinsed in deionized water and placed in a working phosphotungstic/ phosphomolybdic acid solution (Sigma HT15-2, HT15-3) for 5 min.  Finally, slides were placed in 1% acetic acid, for 2 min, rinsed in water, dehydrated through alcohol, cleared in xylene and mounted.

# CD31 immunohistochemistry

Determination of micro-vessel density was performed as follows. Briefly, following deparaffinisation and antigen retrieval, CD31 levels were detected by incubating tissue sections with anti-CD31 (goat polyclonal IgG, sc-1506 (M-20), Santa Cruz Biotechnlogies, Santa Cruz, CA, USA, Antibody Registry number AB_2161037) followed by detection with a biotinylated secondary antibody (Vector Laboratories Ltd, Peterborough, UK), ABC detection system (Vector Laboratories) and DAB chromogen.  Sections were counterstained with haematoxylin. Quantification was performed using an in-house image segmentation algorithm designed for immunohistochemical (IHC) microvessel images. Segmentation of IHC images is challenging in general due to a number of issues, e.g., inter-image/intra-image variability, non-specific/background staining, artefacts. In the case of IHC micro-vessel images, there is an additional issue – incompletely or weakly stained vessels. Another issue which presented itself during testing on study images was the presence of tears in the tissue, which would falsely be identified as vessels. Matlab 7.11.0 software (MathWorks, Natick, MA, USA) was used for extensive experimentation and development of the algorithm. Segmentation outputs from the analyses of representative CD31 images are shown in Supplemental Figure 1.

# Proteomic Analysis: Sample Preparation and Mass Spectrometry (MS)

In order to facilitate analysis of an extended dynamic range of proteins, extracts from murine myocardial tissue was fractionated into a cytosolic (soluble) fraction and a crude membrane (insoluble) fraction in the following manner: Snap frozen whole organs were ground to a powder on dry ice, transferred to a tissue grinder in 500 µL of ice cold lysis buffer (40 mM Tris pH 8.8, 600 mM NaCl, protease inhibitor and Benzonase) and mechanically homogenized on ice. Lysate was transferred to 1.5 mL Beckman tube with glass Pasteur pipette and grinder rinsed with an additional 500 µL of ice cold lysis buffer. Samples were ultracentrifuged at 100,000 *g* for 1 hour at 10 °C (Beckman Optima TLX Ultracentrifuge, Rotor TLA 55, Indianapolis, IN, USA) and the supernatant containing the soluble protein fraction collected and stored at -80 °C (cytosolic fraction). The remaining pellet containing the insoluble protein fraction was twice re-suspended in 1 µL of 1 M KCl followed by centrifugation step at 50,000 *g* for 1 hour at 10 °C (Haereus Centrifuge 28RS Rotor 3740, Waltham, MA, USA), after which the pellet was washed twice with 1 µL of 100 µM $Na_2CO_3$ and centrifuged at 50,000 *g* for 1 hour at 10 °C. The resulting pellet was dissolved in 200 µL extraction buffer (8M urea, 1% 3-[(3-cholamidopropyl)dimethylammonio]-1-propanesulfonate (CHAPS), 50 mM Tris, pH8.8) and samples were then vortexed and shaken for 1 hour and sonicated until sample

appeared to be broken up, with no visible clumps. The samples were then centrifuged at room temperature at 21,000 *g* for 15 min at maximum speed and supernatant collected.

Cytosolic and membrane proteins (100 µg and 20 µg respectively) were reduced and alkylated using a 3-fold excess of dithiothreitol and a 6-fold excess of iodoacetamide by weight. Cytosolic proteins were then digested overnight at room temperature using 2 µg trypsin (sequencing grade, Promega, Madison, WI, USA). Resulting peptides were desalted using solid phase extraction (Strata SDB-L Styrene Divinylbenzene columns containing 25 µg of 100 µm / 260Å beads, (Phenomenex, Torrance, CA, USA ), washed twice with 1.0 % v/v formic acid, and eluted with 250 µL of 70% v/v acetonitrile, 1.0 % v/v formic acid and dried overnight in a speed-vac concentrator (Eppendorf, Hamburg, Germany). Proteins from the crude membrane fraction (10 µg) were further fractionated by SDS-PAGE (10% SDS PAGE; TAE (40 mM Tris-Acetate and 1 mM $Na_2EDTA$) buffer system; Invitrogen, Waltham, MA, USA). After protein staining with colloidal blue, 5 bands (270-120 kDa, 120-55 kDa, 55-28 kDa, 28-17 kDa, 17-8 kDa as estimated by comparing with the molecular mass marker) were excised and subjected to in-gel tryptic digestion as follows. Each acrylamide piece was cut in pieces of approx. 1 $mm^2$ and transferred to an Eppendorf tube. The gel pieces were de-stained with 50% acetonitrile (in water) for 10 min, then with de-staining solution (100 mM ammonium bicarbonate, 30% v/v acetonitrile) until completely colourless. The acrylamide was then dehydrated with 100% acetonitrile for 15 min after which the bands were dried in a Speed Vac for 10 min without heating. Digestion was carried out in 50 µL of tryptic digestion buffer (40 mM ammonium bicarbonate, 10 ng/mL trypsin (Promega)) overnight at room temperature, after which the supernatant was collected. The supernatants were then dried in a Speed Vac without heating and the peptide extract was kept at -80 °C until further use.

Peptide samples were analysed by nanoscale LC-ESI-MS/MS using an Ultimate 3000 nanoflow chromatographic system (Thermo Electron, Waltham, MA, USA) coupled to a LTQ-Orbitrap tandem mass spectrometer (Thermo Electron) equipped with a nanoelectrospray ion source. Peptide separation was achieved using an analytical fused silica emitter (75/360 µm i.d./o.d., tip diameter 8±1 mm, New Objective home-packed at 400 bar with 15 - 19 cm of 3 mm Reprosil C18 A.Q. reverse phase material (Dr. Maisch)).

The peptide mixture of each sample was dissolved in 100 µL of buffer A (2% v/v acetonitrile, 0.5% v/v acetic acid) and approximately 250 ng equivalent of the peptide mixture were injected into the system at a flow rate of 450 nL/min at 100 % buffer A for 12 min. After loading, the flow was decreased to 250 nL/min and peptides were eluted from the reverse phase column as follows: 12-14 min, 0-5% buffer B (80% v/v acetonitrile, 0.5% v/v acetic acid); 14-30 min, 5-30% buffer B using the curve 4 (slightly concave) of the Chromoleon software (Thermo Electron); 30-90 min, 30-55% buffer B using the curve 6 (slightly convex) of the Chromeleon software. The column was then washed for 15 min with 100% buffer B at 350 nL/min and re-equilibrated for 25 min in 100% buffer A at 350 nL/min.

Peptides were analysed by tandem MS (standard operating parameters of electrospray voltage = 2.0 kV; capillary temperature = 170°C). Survey scans (scanning range m/z 400-1650) were recorded in the Orbitrap mass analyser at a resolution of 30,000 with the lock mass option enabled. Data-dependent MS/MS spectra of the five most abundant ions from the survey scan were recorded in the LTQ ion trap using a normalised collision energy of 32 % for MS/MS (30 ms activation, q = 0.25) and a selection threshold of 500. Target ions selected for MS/MS were dynamically excluded for 30 seconds.

# Mass Spectrometry Data Processing

Raw mass spectrometry data from crude membrane and soluble fraction were separately processed using the SEQUEST search algorithm (SEQUEST version 27.0, revision 12, Thermo Electron) and searches were performed using the UniProtKB/Swiss-Prot protein knowledgebase database 2011_07 (23927 "*Mus musculus*" sequences concatenated with their decoy entries). Data were searched with a mass tolerance of +/-5 ppm for parent ions and +/-1.0 Da for fragment ions. Methionines

(reduced/oxidized; +15.9949 Da) were considered as differential modifications while cysteines were considered as fully carbamidomethylated (+57.0199 Da). Only fully tryptic peptides with no more than one miscleavage were considered for data analysis. The false discovery rate (FDR) of the identified proteins was evaluated using an in-house Roche algorithm[3] with only proteins with a FDR < 1% included for further analysis.

In analysing mass spectrometry data (Supplemental Figure S3), only proteins with at least two unique peptides in one experimental condition and present in a minimum of two experimental conditions were processed further. Identified proteins were next normalised in the following manner. The relative protein abundance was determined by taking the product of the peptide count, coverage and the median mass of all identified proteins and divided by each individual protein's mass. Samples were then ranked according to their relative abundance and log transformed for comparison between conditions. Significantly changed proteins following sunitinib treatment were reported by a Mann Whitney U-test (SPSS, IBM Corporation, Armonk, NY, USA) with a p-value cut-off of 0.05. In order to evaluate the robustness of both the data set and the approach, hierarchical clustering analysis (Spotfire, Somerville, MA, USA) and principle component analysis (PCA) (Partek Genomics Suite, St. Louis, MO, USA) were performed. Parameters of hierarchical clustering analysis were as follows: complete linkage (clustering method), average value (ordering weight), Z-score calculation (normalization) for both row and column dendrograms. Median log rank values for each treatment group were calculated in order to determine the difference between treatment conditions. Ingenuity® Pathway Analysis (IPA®, QIAGEN Redwood City, www.qiagen.com/ingenuity) was used in order to identify canonical and toxicity pathways enriched in the data set. IPA generated networks based on their connectivity with a data sets and a score was assigned by the software. The score indicates network relevance to the proteins in the input dataset. The intensity of the colour in each network indicates the degree of up- (red) or down- (green) regulation.

# Proteomic Validation Experiments: Immunoblot and ELISA Assays

For confirmation of proteomic data generated, heart lysates prepared for mass spectrometry analysis were analysed by Western blot. Briefly, 100 μg total lysates were separated on 10% SDS-PAGE gels under reducing conditions. Proteins were then transferred using a semi-dry approach. Specific proteins were then detected using the following antibodies; hypoxia-inducible factor 1 alpha (HIF1α) (Purified Mouse Anti-Human HIF-1α Clone 54/HIF-1α (RUO), Becton Dickinson, Franklin Lakes, NJ, USA, Immunogen: Human HIF-1α aa. 610-727), phosphorylated-Acetyl co-enzyme A carboxylase (phospho-ACC) (polyclonal rabbit, #3661, Cell Signalling Technologies, Danvers, MA, USA, Antigen used: Ser79 of rat ACC, Antibody registry number AB_2288840), succinate dehydrogenase complex, subunit A (SDHA) (polyclonal rabbit, ab137756, Abcam Inc., Cambridge, MA, USA, Recombinant fragment, corresponding to a region within amino acids 12-234 of Human SDHA), hydroxyacyl-Coenzyme A dehydrogenase/3-ketoacyl-coenzyme A thiolase/enoyl-coenzyme A hydratase (trifunctional protein), alpha subunit (HADHA) (polyclonal rabbit, ab54477, Abcam Inc. Antibody Registry number AB_2263836 and CD36 (polyclonal rabbit, ab64014, Abcam Inc. Antibody Registry number AB_10672537). HRP-labelled anti-mouse or anti-rabbit secondary antibodies were then used and after substrate addition blots were developed using chemiluminescence and x-ray film. Images were scanned and densitometry was performed using Image J software. For detection of mitochondrial complex 1 level in mouse heart tissue a specific enzyme-linked immunosorbent assay (ELISA) (ab136809, Abcam Inc) was performed following the manufacturer's instructions. The capture antibody (Clone: 20D1AB7) used in this assay detects whole Complex 1. Briefly, 100 μg total protein was loaded per well in duplicate. Plates were read at 600 nm. Data was analysed using Microsoft Excel.

## Statistical analyses

Animal studies: To compare inter-group differences, data were averaged within each group and compared using two-tailed independent t-tests at each time point and two-way ANOVA for overall comparisons. For intra-group changes, grouped averages of pre-treatment measurements were compared to those at subsequent time points using two-tailed paired t-tests (GraphPad Prism version 5.01 for Windows, GraphPad Software, San Diego CA, USA, www.graphpad.com).

For Mitochondrial complex 1 ELISA, TUNEL assay and anti-CD31 staining, two-tailed independent t-tests were used to determine the difference between groups, with data presented as values ± SEM. For lipid droplet quantification (EM), Image J software was used to count the number of lipid droplets visible in a 625 $\mu m^2$ field (× 6500 original magnification) and data presented as means ± SEM. For ORO staining, one-way ANOVA followed by the least significant difference calculation was used to analyse differences between groups and all values are shown as means ± SD.

LC/MS-MS: Significantly changed protein levels following treatment were assessed by Mann Whitney U-test with a p value cut-off of 0.05. To evaluate the robustness of both the data set and the approach, hierarchical clustering and principal component analysis (PCA) were performed. Median log rank values for each treatment group were calculated to determine treatment differences. Ingenuity® Pathway Analysis (IPA®, QIAGEN Redwood City, www.qiagen.com/ingenuity) was used to identify enriched canonical and toxicity pathways.

## References

1. Patlak CS, Blasberg RG, Fenstermacher JD. Graphical evaluation of blood-to-brain transfer constants from multiple-time uptake data. Journal of cerebral blood flow and metabolism : official journal of the International Society of Cerebral Blood Flow and Metabolism. 1983;3(1):1-7. Epub 1983/03/01. doi: 10.1038/jcbfm.1983.1. PubMed PMID: 6822610.
2. Tantawy MN, Peterson TE. Simplified [18F]FDG image-derived input function using the left ventricle, liver, and one venous blood sample. Molecular imaging. 2010;9(2):76-86. Epub 2010/03/20. PubMed PMID: 20236605.
3. Berntenis N, Kux van Geijtenbeek S, Ducret A. A novel Protein Scoring Algorithm for shotgun proteomics (Poster presented at HUPO Congress; poster No. C522). 2009.
