# Supplementary material for: A Novel Positron Emission Tomography (PET) Approach to Monitor Cardiac Metabolic Pathway Remodeling in Response to Sunitinib Malate
Source: PLoS One. 2017 Jan 27;12(1):e0169964. doi: 10.1371/journal.pone.0169964 (PMC5271313; doi:10.1371/journal.pone.0169964)
Supplement: S1 Fig — (PDF) [file pone.0169964.s002.pdf]

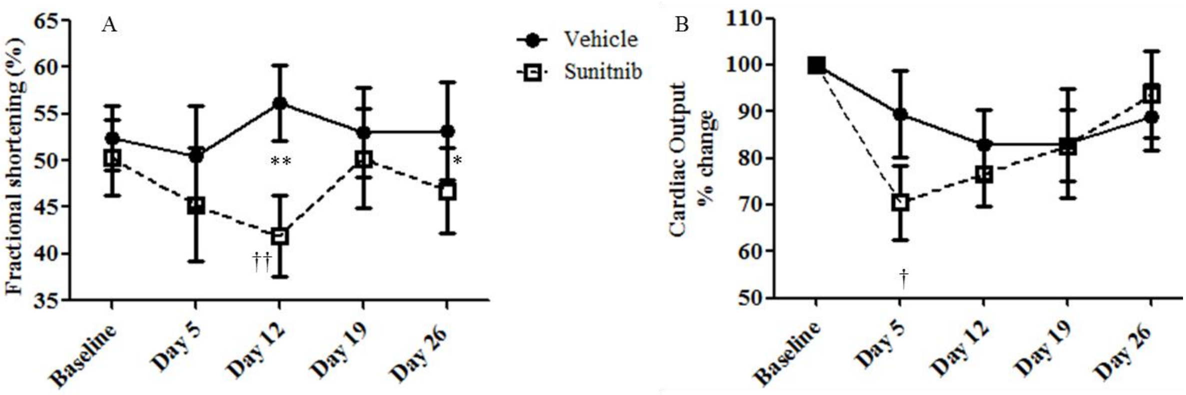

S1 Fig. The effect of sunitinib on A) fractional shortening and B) cardiac output (CO) in rats. (n = 12, significant difference between groups (unpaired t-test, *p<0.05 **p<0.01), Significant change from pre-treatment values (paired t-test, †p<0.05 ††p<0.01). Error bars = SEM
