# Supplementary material for: A Novel Positron Emission Tomography (PET) Approach to Monitor Cardiac Metabolic Pathway Remodeling in Response to Sunitinib Malate
Source: PLoS One. 2017 Jan 27;12(1):e0169964. doi: 10.1371/journal.pone.0169964 (PMC5271313; doi:10.1371/journal.pone.0169964)
Supplement: S2 Fig — (PDF) [file pone.0169964.s003.pdf]

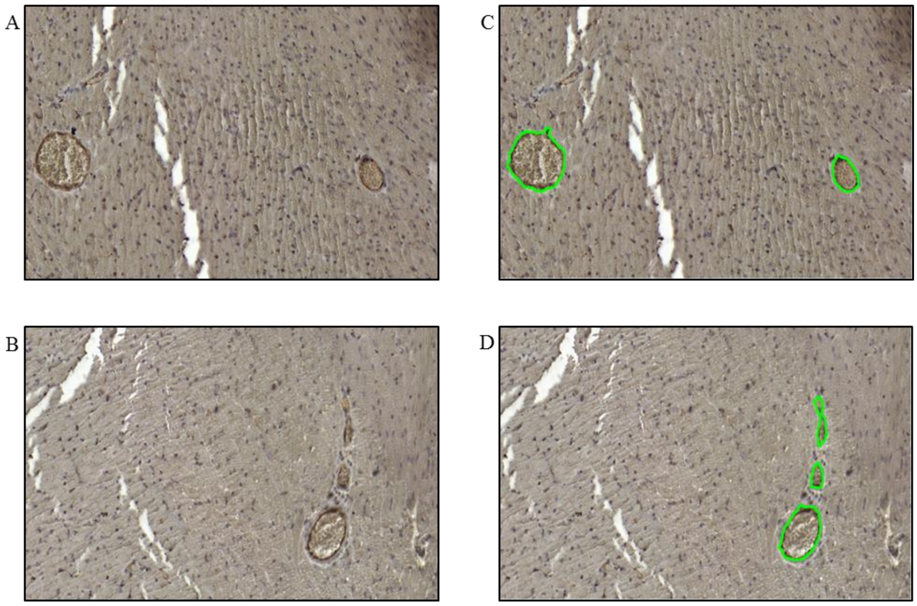


**S2 Fig. CD31 micro-vessel segmentation output.** Test CD31 Immunohistochemical images (A and B). Respective segmentation mark-up images (C and D).
