# Supplementary material for: A Novel Positron Emission Tomography (PET) Approach to Monitor Cardiac Metabolic Pathway Remodeling in Response to Sunitinib Malate
Source: PLoS One. 2017 Jan 27;12(1):e0169964. doi: 10.1371/journal.pone.0169964 (PMC5271313; doi:10.1371/journal.pone.0169964)
Supplement: S3 Fig — (PDF) [file pone.0169964.s004.pdf]

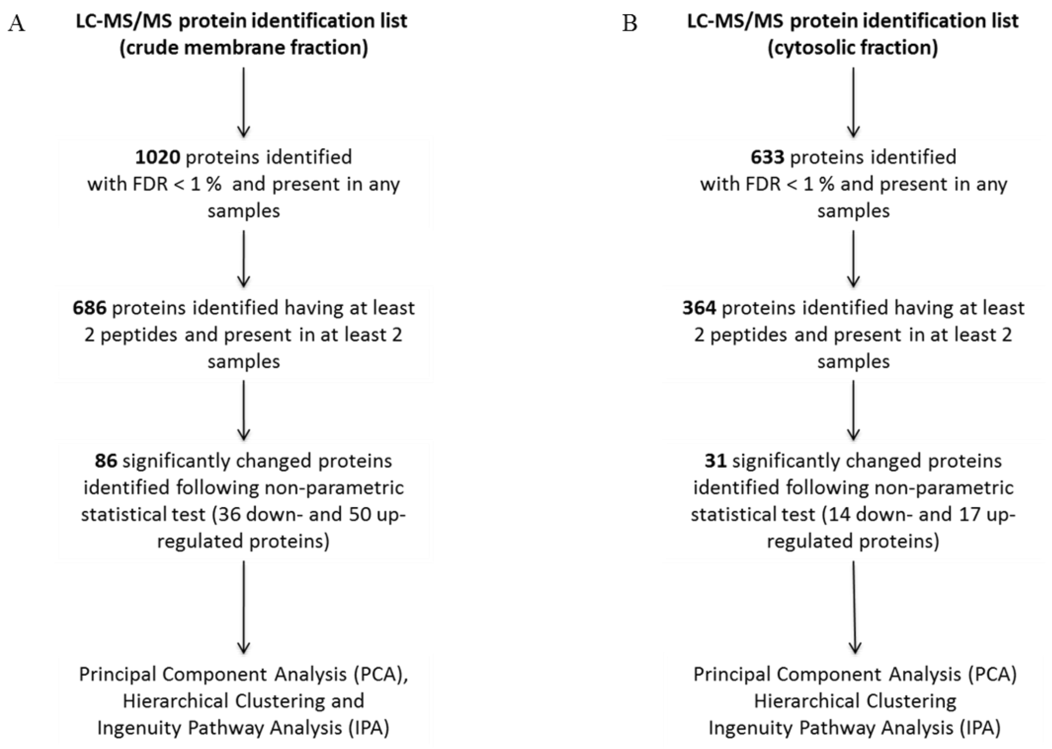


**S3 Fig. LC-MS/MS data analysis of insoluble and soluble fractions.** LC-MS/MS data analysis of insoluble (A) and soluble (B) fractions identified 1020 crude membrane proteins and 633 cytosolic proteins with FDR < 1 % respectively. 686 crude membrane proteins and 364 cytosolic proteins were identified which had at least two unique peptides in one experimental condition and were present in at least two experimental conditions. Following ranking and log transformation 86 (36 down- and 50 up-regulated) and 31 (14 down- and 17 up-regulated) significantly changed proteins respectively for crude membrane and cytosolic fractions were identified in sunitinib treated versus vehicle control.
