# Supplementary material for: A Novel Positron Emission Tomography (PET) Approach to Monitor Cardiac Metabolic Pathway Remodeling in Response to Sunitinib Malate
Source: PLoS One. 2017 Jan 27;12(1):e0169964. doi: 10.1371/journal.pone.0169964 (PMC5271313; doi:10.1371/journal.pone.0169964)
Supplement: S4 Fig — (PDF) [file pone.0169964.s005.pdf]

Hierarchical clustering and PCA were performed separately for each fraction (Figure S4) in order to demonstrate different protein expression profile of heart tissue derived from animals treated with vehicle or sunitinib 40 mg/kg. Hierarchical clustering demonstrated similarities among treatment groups where animals within a treatment group clustered together in both fractions (Figure S4 C/D) Moreover, PCA results also confirmed different protein expression pattern between treatment groups. The eight heart samples analysed here were clearly separated into two sets, one set with four control heart samples and the other set with four sunitinib-treated heart samples (Supplemental Figure 4 A/B). These findings indicate the robustness of both the analysis approach taken and the data despite the limited sample size.

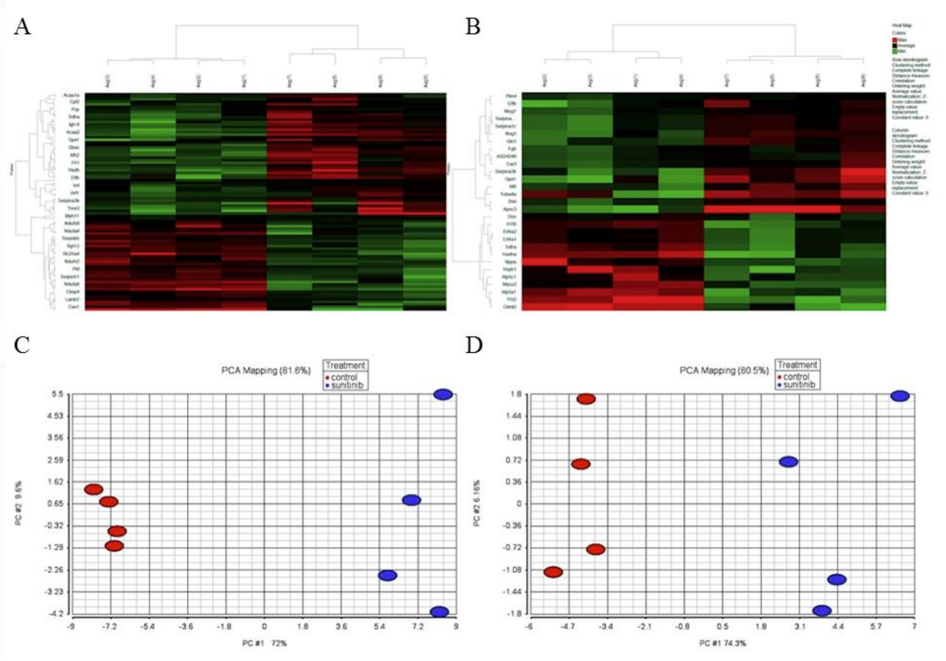

S4 Fig. Hierarchical clustering and principle component analysis. Supervised hierarchical clustering (A/B) and principle component analysis (PCA) (C/D) of significantly ($p < 0.05$, Mann Whitney U-test) altered proteins from insoluble (A/C) and soluble (B/D) fractions of heart tissue were grouped. In (A/B), red lines represent proteins whose expression levels were up-regulated, green lines represent down-regulated proteins, and black lines represent unchanged proteins relative to the group (control (mice # 1 - 4) and sunitinib (mice # 5 - 8)). In (C/D), the protein expression profiles of heart tissues derived from control mice (n = 4, red) and sunitinib-treated mice (n = 4, blue) were compared using PCA. The two-dimensional (2D) plot views of protein expression data are shown, with respect to their correlation.
