# Supplementary material for: A Novel Positron Emission Tomography (PET) Approach to Monitor Cardiac Metabolic Pathway Remodeling in Response to Sunitinib Malate
Source: PLoS One. 2017 Jan 27;12(1):e0169964. doi: 10.1371/journal.pone.0169964 (PMC5271313; doi:10.1371/journal.pone.0169964)
Supplement: S1 Table — (PDF) [file pone.0169964.s006.pdf]

S1 Table. Significantly changed proteins (86) identified by LC-MS/MS in the crude membrane fraction calculated by Log^2 relative ranks comparing expression in sunitinib versus vehicle myocardial tissue.

Significantly Changed Proteins identified by LC-MS/MS in Crude Membrane Fraction		
Accession Number	Protein Symbol	Difference in log2 relative ranks (sunitinib-control)
Q9CQD1	Rab5a	-1.198
Q8R1M2	H2afj	-1.662
P62270	Rps18	-1.352
P47963	Rpl13	-0.730
P62075	Timm13	-0.627
P48962	Slc25a4	-0.696
P62264	Rps14	-0.652
P35278	Rab5c	-0.592
Q9CQZ5	Ndufa6	-0.749
Q9D6J6	Ndufv2	-0.469
Q9CQB5	Cisd2	-0.797
Q9CQ69	Uqcrq	-0.617
P19324	Serpinh1	-0.572
P63028	Tpt1	-0.577
P49817	Cav1	-0.573
Q91VC9	Ghitm	-0.447
Q99P58	Rab27b	-0.529
Q8BP67	Rpl24	-0.486
Q8R1V4	Tmed4	-0.503
O54724	Ptrf	-0.427
Q9CQZ6	Ndufb3	-0.481
P52503	Ndufs6	-0.314
P09055	Itgb1	-0.348
Q925I1	Atad3a	-0.315
Q9JKL4	Ndufaf3	-0.366
Q9CPQ1	Cox6c	-0.453
Q8R5J9	Arl6ip5	-0.289
Q4VAE3	Tmem65	-0.273
Q8BMK4	Ckap4	-0.165
Q3UIU2	Ndufb6	-0.560
P97450	Atp5j	-0.608
Q9DCC8	Tomm20	-0.198
O88322	Nid2	-0.152
Q8BGY7	4933403F05Rik	-0.044
Q61292	Lamb2	-0.056
Q62425	Ndufa4	-0.212
O08638	Myh11	0.088
Q8CHT0	Aldh4a1	0.094
P29758	Oat	0.160
Q99KI0	Aco2	0.187
O09161	Casq2	0.194
Q8VEH3	Arl8a	0.123
P52825	Cpt2	0.219
Q9JHI5	Ivd	0.295
Q9EQ20	Aldh6a1	0.353
P27573	Mpz	0.330
P07759	Serpina3k	0.374
Q61838	Pzp	0.334
P05202	Got2	0.393
Q921H8	Acaa1a	0.366
Q8VCH0	Acaa1b	0.366
P07758	Serpina1a	0.414
Q00896	Serpina1c	0.414
P05201	Got1	0.372
P49222	Epb4.2	0.489
Q9QYR9	Acot2	0.531
P84078	Arf1	0.499
P61205	Arf3	0.503
Q9CQQ7	Atp5f1	0.585
P47738	Aldh2	0.543
Q8K2B3	Sdha	0.623
Q99LC5	Etfa	0.619
P45952	Acadm	0.590
Q8VCT4	Ces1d	0.731
Q9D0K2	Oxct1	0.762
O55126	Gbas	0.716
Q8BWM0	Ptges2	0.729
Q8BWT1	Acaa2	0.809
Q6P8J7	Ckmt2	0.759
P08249	Mdh2	0.823
P01872	Igh-6	0.770
P51174	Acadl	0.810
Q8QZT1	Acat1	0.875
P48787	Tnni3	0.909
Q9DCM2	Gstk1	0.834
Q99JY0	Hadhb	0.939
P54071	Idh2	0.887
Q9CQA3	Sdhb	0.916
Q9DCW4	Etfb	0.955
P97807	Fh1	1.016
P11352	Gpx1	0.928
Q8BMS1	Hadha	1.213
P58771	Tpm1	1.092
O55143	Atp2a2	1.074
Q61425	Hadh	1.235
Q02566	Myh6	1.175
