# Supplementary material for: A Novel Positron Emission Tomography (PET) Approach to Monitor Cardiac Metabolic Pathway Remodeling in Response to Sunitinib Malate
Source: PLoS One. 2017 Jan 27;12(1):e0169964. doi: 10.1371/journal.pone.0169964 (PMC5271313; doi:10.1371/journal.pone.0169964)
Supplement: S2 Table — (PDF) [file pone.0169964.s007.pdf]

S2 Table. Significantly changed (31) proteins identified by LC-MS/MS in the cytosolic fraction calculated by Log^2 relative ranks comparing expression in sunitinib versus vehicle myocardial tissue

Significantly Changed Proteins Identified by LC-MS/MS in Cytosolic Fraction		
Accession Number	Protein Symbol	Difference in log2 relative ranks (sunitinib-control)
O70433	Fhl2	-1.300
Q8BMS1	Hadha	-1.069
Q00896	Serpina1c	0.479
P07758	Serpina1a	0.480
Q9D2G2	Dlst	0.326
P10922	H1f0	-0.525
O88492-2	Plin4	0.158
P33622	Apoc3	1.485
Q8K2B3	Sdha	-0.684
Q6P8J7	Ckmt2	-1.226
P28665	Mug1	0.262
Q03265	Atp5a1	-0.860
O08677-2	Kng1	0.553
P60843	Eif4a1	-0.335
P10630	Eif4a2	-0.327
Q9JJW5	Myoz2	-0.597
Q9CPU0	Glo1	0.580
P11352	Gpx1	1.072
P07759	Serpina3k	0.791
P14602-2	Hspb1	-0.740
O88492	Plin4	0.112
Q8K0E8	Fgb	0.239
P14602	Hspb1	-0.676
P16015	Car3	0.240
Q91VR2	Atp5c1	-0.438
P34884	Mif	0.505
P05125	Nppa	-0.471
Q9DCW4	Etfb	0.301
P31001	Des	-0.209
P68368	Tuba4a	1.063
P03987	AI324046	0.169
